# In Silico Development of Novel Quinazoline-Based EGFR Inhibitors via 3D-QSAR, Docking, ADMET, and Molecular Dynamics

**DOI:** 10.3390/ijms27021050

**Published:** 2026-01-21

**Authors:** Mohamed Moussaoui, Soukayna Baammi, Mouna Baassi, Said Kerraj, Hatim Soufi, Younes Rachdi, Mohammed El Idrissi, Mohammed Salah, Mohammed Elalaoui Belghiti, Rachid Daoud, Said Belaaouad

**Affiliations:** 1 Laboratory of Physical Chemistry of Applied Materials (LCPMA), Faculty of Sciences Ben M’Sick, Hassan II University of Casablanca, Casablanca 20670, Morocco; mounabaassi1993@gmail.com (M.B.); krjsaid@gmail.com (S.K.); rachdi.smc@gmail.com (Y.R.);; 2 Chemical and Biochemical Sciences-Green Processing Engineering, Mohammed VI Polytechnic University, Ben Guerir 43150, Morocco; soukayna.baammi@um6p.ma; 3 Chemical Processes and Applied Materials Team, Faculty Polydisciplinary Sultan Moulay Slimane University, Beni-Mellal 23000, Morocco; 4 Team of Chemoinformatics Research and Spectroscopy and Quantum Chemistry, Department of Chemistry, Faculty of Science, University Chouaib Doukkali, B. P. 20, El Jadida 24000, Morocco

**Keywords:** 3D-QSAR, CoMFA, CoMSIA, ADMET, molecular docking, quinazoline, molecular dynamic, EGFR inhibitors, lung cancer, anticancer

## Abstract

A library of thirty-one quinazoline derivatives was assessed as potential inhibitors of epidermal growth factor receptor kinase (EGFR) using 3D-QSAR methods, namely Comparative Molecular Field Analysis (CoMFA) and Comparative Molecular Similarity Indices Analysis (CoMSIA). Training and test sets were generated by aligning the molecules to the lowest-energy conformer of the most active compound. The optimized models exhibited strong statistical performance, with R^2^ values of 0.981 (CoMFA) and 0.978 (CoMSIA), and cross-validation coefficients (Q^2^) of 0.645 and 0.729, respectively. External validation confirmed their predictive power, yielding R^2^ values of 0.929 and 0.909. Guided by these models, eighteen new quinazoline candidates were designed and evaluated for drug likeness and ADMET (Absorption, Distribution, Metabolism, Excretion, and Toxicity) properties using in silico approaches. Molecular docking and molecular dynamics simulations highlighted the binding features and stability of these derivatives, with compound Pred65 demonstrating superior affinity and stability compared to Erlotinib. Collectively, the study provides valuable insights for the optimization of quinazoline scaffolds as EGFR inhibitors, supporting the development of promising anticancer leads.

## 1. Introduction

Lung cancer remains one of the most prevalent and lethal cancers worldwide, with non-small cell lung cancer (NSCLC) accounting for the majority of diagnosed cases [1]. Despite the introduction of chemotherapy, radiotherapy, and targeted agents, treatment options remain limited, particularly in advanced stages [2]. Among the molecular pathways implicated in NSCLC, the epidermal growth factor receptor (EGFR) has emerged as a key therapeutic target due to its involvement in cell proliferation, differentiation, and survival [3]. Small-molecule EGFR tyrosine kinase inhibitors, such as Erlotinib, have been successfully employed in clinical practice [4]. However, issues such as drug resistance and modest binding affinity limit their long-term clinical benefit [5].

To overcome these limitations, there is an urgent need to develop new EGFR inhibitors with enhanced potency, stability, and selectivity [6]. Computational methodologies offer a powerful framework for this purpose, allowing rapid evaluation of molecular interactions, prediction of pharmacokinetic properties, and assessment of complex stability prior to experimental validation [7]. In this context, the present work focuses on quinazoline derivatives, a well-known scaffold for kinase inhibition, to identify novel candidates against EGFR. In this regard, integrated computational approaches have become essential tools in contemporary drug discovery [8], enabling efficient exploration of structure-activity relationships and rational prioritization of lead candidates prior to experimental validation.

The novelty of the present study lies in the rational integration of multiple complementary in silico techniques to guide the design and prioritization of quinazoline-based EGFR inhibitors. Rather than relying on a single computational method, this work combines statistically validated 3D-QSAR modeling (CoMFA and CoMSIA) with contour-map-driven molecular design, comprehensive ADMET evaluation, molecular docking, and molecular dynamics simulations.

This multi-level computational framework enables the identification of key structural determinants underlying EGFR inhibition and supports the systematic prioritization of newly designed compounds as promising computational lead candidates. Overall, the proposed workflow provides a robust, efficient strategy for early-stage lead optimization before experimental validation.

By applying this integrated approach, we explored the structural features governing inhibitory activity and dynamic stability within the EGFR binding site. The workflow facilitated the identification of promising candidates and highlighted Pred65 as a particularly promising computational lead candidate relative to the reference inhibitor Erlotinib. Collectively, these findings contribute to the rational design and further optimization of quinazoline-based EGFR inhibitors for NSCLC therapy.

## 2. Results and Discussion

### 2.1. Molecular Alignment

**Figure 1:**
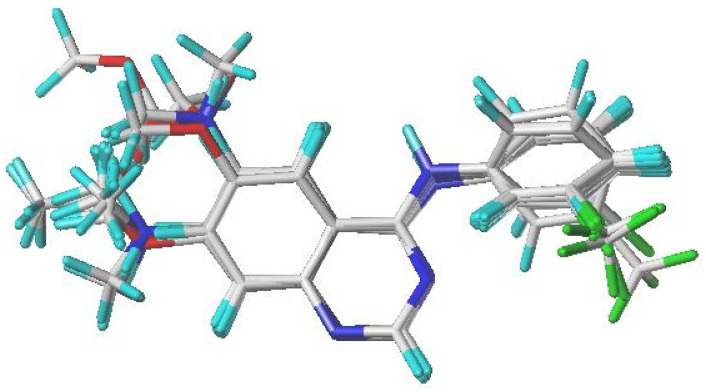
Alignment and superimposition of the 31 investigated quinazoline derivatives, using compound **17** (the most active molecule) as the reference template.

Molecular alignment is a crucial stage in developing reliable 3D-QSAR models. In this study, compound **17**, identified as the most biologically active molecule, was chosen as the reference template for aligning the dataset and for generating the contour maps. As shown in Figure 1, all structures from both the training and test sets were superimposed onto a common scaffold, following an alignment strategy derived from the best docking pose of compound **17**.

### 2.2. CoMFA and CoMSIA Studies

Table 1 summarizes the statistical outcomes obtained from the CoMFA and CoMSIA analyses. In the CoMFA model, steric and electrostatic descriptors accounted for 42.6% and 57.4% of the variance, respectively. The cross-validated correlation coefficient (Q^2^), determined using the leave-one-out (LOO) procedure, was 0.645 with an optimal number of five components. The non-cross-validated determination coefficient (R^2^) reached 0.981, with a standard error of estimate (SEE) of 0.157 and an F-value of 202.418, indicating strong predictive power. External validation confirmed this, with R^2^pred equal to 0.929.

Among the developed models, the CoMSIA/SEHD variant emerged as the most robust, with Q^2^ = 0.729 (6 principal components), R^2^ = 0.978, SEE = 0.173, and an F-test score of 139.580. Complementary results are listed in Table 2, where predicted pIC_50_ values are compared with experimental data, highlighting the descriptive and predictive accuracy of the CoMSIA framework.

In these models, Q^2^ denotes the cross-validated correlation coefficient, N indicates the optimal number of latent components, R^2^ represents the non-cross-validated determination coefficient, SEE refers to the standard estimation error, and F corresponds to the F-test statistic. External predictive ability is evaluated using R^2^_pred_. Descriptor contributions include steric (S), electrostatic (E), hydrophobic (H), hydrogen bond donor (D), and hydrogen bond acceptor (A) fields.

A 3D-QSAR model is generally considered reliable if R^2^, R^2^_pred_, and Q^2^ exceed thresholds of 0.6, 0.6, and 0.5, respectively. Both CoMFA and CoMSIA/SEHD models met these requirements, reflecting high stability and predictive accuracy. Robustness and predictability tests further validated the CoMSIA/SEHD model. This model satisfied all criteria proposed by Roy, Golbraikh, and Tropsha, and provided deeper insight than CoMFA by offering improved predictive capability for novel compounds. Accordingly, CoMSIA/SEHD contour maps were used to define structural features critical for enhancing activity and guiding the design of new active derivatives.

### 2.3. Graphical Representation of the CoMSIA/SEHD Model

**Figure 2:**
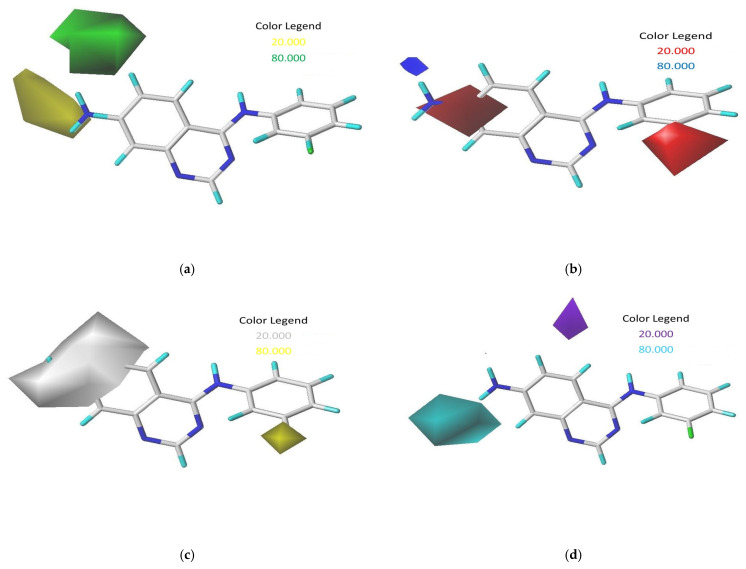
Contour maps derived from CoMSIA analysis with 2 Å grid spacing using compound 17 as a reference: (**a**) steric field, (**b**) electrostatic field, (**c**) hydrophobic field, and (**d**) hydrogen bond donor field.

To interpret the data from the top-performing 3D-QSAR model, we used the CoMSIA/SEHD contour map, referencing compound **17**, which exhibited the highest activity. Figure 2 illustrates the Steric, Electrostatic, Hydrophobic, and Hydrogen bond Donor fields as per the CoMSIA contour maps.

Bulky substituents that enhance biological activity are represented by green contours, which account for an 80% contribution, while areas where such groups are detrimental to activity are marked by yellow contours, representing a 20% contribution, within the steric field. For the electrostatic field, blue contours signify an 80% contribution where positively charged areas are preferred, and red contours show a 20% contribution where negatively charged areas are advantageous. The map also displays hydrophobic interactions, with yellow contours (80% contribution) highlighting regions where hydrophobic characteristics are beneficial, and white contours (20% contribution) indicating the opposite. Additionally, in the hydrogen-bond donor field, cyan contours depict an 80% contribution for regions where hydrogen-bond donor groups are advantageous for activity, and purple contours show a 20% contribution for areas less favorable for such interactions [9].

In the CoMSIA analysis, we noted a significant green contour surrounding R_1_ substitutions in the ortho position of quinazoline compounds (Figure 2a), suggesting the need to select bulky substitution groups in this area. Similarly, in the CoMSIA electrostatic contour maps (Figure 2b), a blue contour was observed adjacent to R2 substitutions in the meta position for quinazoline compounds, indicating that incorporating highly electropositive groups or atoms in this position could enhance activity. This observation is supported by the higher activity of compound **17**, which features an NH2 group at the meta position, compared to compounds **2** and **23**, which have -H and -NO_2_ in the same position. Yellow and white contours in hydrophobic fields help identify regions preferring hydrophobic and hydrophilic properties, respectively. The presence of a yellow contour around the R_3_ chain in Figure 2c suggests the advantage of a hydrophobic substituent for inhibitory activity, while a large white contour at the extremity of R_1_ and R_2_ implies a preference for a hydrophilic group in those regions. Additionally, the cyan outline indicates favorable hydrogen-bond donor groups at the hydrogen-bond site, as depicted by the cyan contour surrounding the NH2 group in Figure 2d, suggesting that hydrogen-bond donors may enhance inhibitory activity, as exemplified by the NH_2_ group functioning as a hydrogen-bond donor in this position.

### 2.4. Design of New Drug Candidates

**Figure 3:**
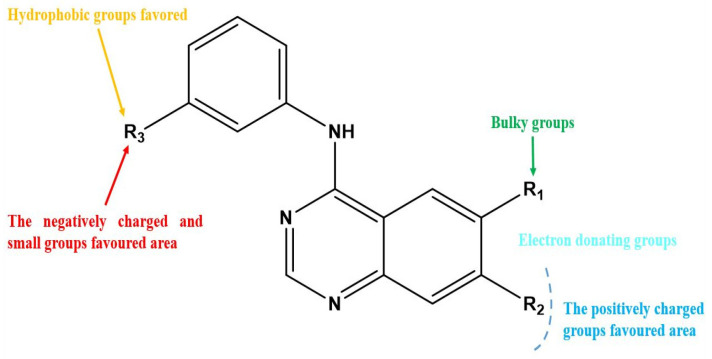
Structure–activity relationship information obtained from the CoMSIA/SEHD model.

After interpreting the results of the 3D-QSAR model and identifying the descriptors that strongly influenced inhibitory activity through map analysis, modifications were made to active molecule 17 by substituting R_1_, R_2_, and R_3_. Utilizing molecule 17 as a template, the newly projected ligands were refined and aligned with the database. These potential medications exhibited greater biological activities than molecule 17. Table 3 illustrates the structure and activity of each predicted molecule, while Figure 3 outlines the strategy employed for designing the new molecules.

### 2.5. Drug-likeness Assessment and ADMET Predictions

When assessing the drug likeness of the newly designed compounds (Pred65-Pred98), their physicochemical characteristics were carefully examined against standard drug filters, including Lipinski’s Rule of Five (RO5), the Veber rule, and the Egan rule. Only ligands that complied with these criteria without violations were advanced for docking simulations. According to Lipinski’s guidelines, a compound is considered drug-like if it has a molecular weight (MW) below 500 g/mol, a partition coefficient (log P) less than 5, no more than 5 hydrogen bond donors (NHD), and fewer than 10 hydrogen bond acceptors (NHA) (Table 4).

It should be acknowledged that some designed compounds exhibit relatively high log P values, which may negatively impact aqueous solubility and oral bioavailability. These physicochemical limitations represent common challenges in early-stage drug discovery and highlight the need for further optimization.

The ADMET pharmacokinetic parameters of the proposed new compounds were assessed using pkCSM [10], an online tool, to confirm their suitability. The outcomes, outlined in Table 5, encompass crucial computed ADMET properties for the top eighteen screened compounds. According to the table, the intestinal absorption rates for these compounds range from 87.034% to 100%, indicating notably high absorption characteristics [11], with Pred86 demonstrating the highest value.

A low Volume of Distribution at Steady State (VDss) is characterized by values under 0.71 L/kg, which translates to a logarithmic value (log VDss) below −0.15. On the other hand, a VDss is deemed high if it surpasses 2.81 L/kg, corresponding to a log VDss value that is above 0.45 [12].

Within the framework of assessing compounds for blood–brain barrier (BBB) and central nervous system (CNS) permeability, specific values serve as thresholds. A LogBB value lower than −1 suggests inadequate brain penetration, while a LogBB greater than 0.3 suggests effective BBB penetration. Additionally, a LogPS value higher than −2 is indicative of sufficient CNS penetration, whereas a LogPS below −3 suggests limited CNS access [13,14]. Consequently, it can be inferred that the compounds under consideration are unlikely to cross these barriers.

Enzymatic metabolism is the biochemical conversion of drugs by the body, essential for transforming drug compounds. Drugs in the body produce various enzymatic metabolites that are key in facilitating reactions across different drug concentrations [15]. It’s critical to grasp the nuances of drug metabolism, as metabolites can vary significantly in their physicochemical and pharmacological characteristics. The Cytochrome P450 (CYP450) enzyme system is particularly important in the initial phase of drug metabolism, known as phase 1 metabolism (oxidation), which our study highlights. Humans have 57 known CYP genes spread across 17 families, yet only the CYP1, CYP2, CYP3, and CYP4 families are involved in metabolizing drugs. Of these, the enzymes CYP1A2, 2C9, 2C19, 2D6, and 3A4 are responsible for the phase I metabolism of more than 90% of drugs [15,16]. Notably, CYP3A4 is identified as the most prominent in our research [17]. The newly designed compounds in our study were found to be both substrates and inhibitors of CYP3A4.

Several designed compounds were predicted to inhibit CYP3A4, which may raise concerns regarding potential drug–drug interactions. However, such predictions primarily serve as early-stage alerts in drug discovery rather than definitive clinical liabilities.

CYP-related risks can often be mitigated through structural optimization, dose adjustment, or formulation strategies during later development stages. Therefore, the observed CYP3A4 inhibition highlights optimization priorities rather than disqualifying these compounds as potential leads.

Clearance serves as a metric to indicate how quickly medications are removed from the body in relation to their internal concentrations [18]. As shown in Table 5, none of the new compounds exhibit any concerns regarding drug persistence. In the early stages of drug development, it’s vital to assess the toxicity of predicted compounds. Thus, every new drug candidate examined in this study undergoes a toxicity evaluation using the AMES test [19]. As indicated in Table 5, all the designed ligands were found to be non-toxic.

Finally, the predicted results (Table 5) indicate that these compounds possess a favorable ADMET profile and exhibit good drug likeness.

### 2.6. Molecular Docking Study

**Figure 4:**
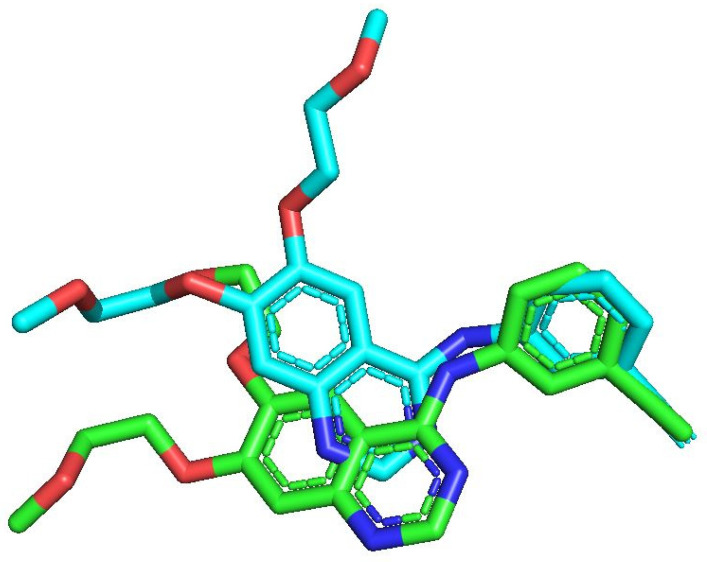
Re-docking validation showing an RMSD of 1.2 Å, with the native ligand represented in green and the redocked pose displayed in blue.

**Figure 5:**
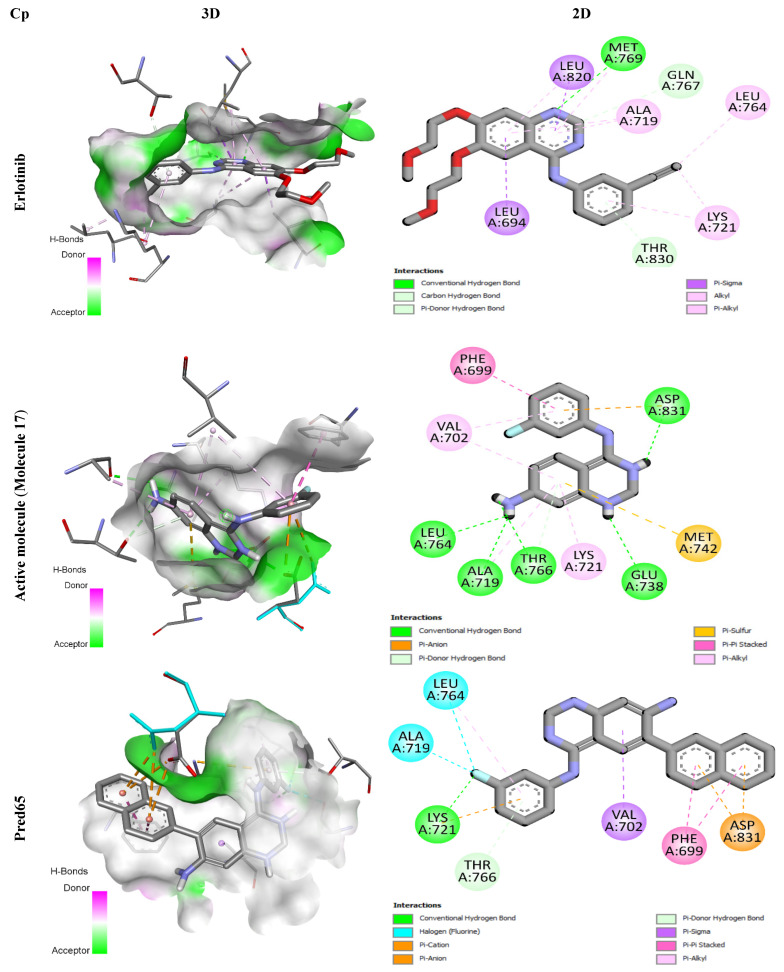
3D and 2D Representations of Binding Interactions for the Three Complex Compounds.

In this work, the binding interactions of the newly designed inhibitors with EGFR were investigated through molecular docking and compared against the reference inhibitor Erlotinib. To validate the docking protocol, the native ligand of EGFR (PDB ID: 1M17) was redocked into its active site, yielding a conformation nearly identical to the crystallographic pose, with an RMSD of 1.2 Å (Figure 4) [20]. Once the docking protocol was confirmed, the same parameters were applied to all compounds, including the reference, the most active molecule from the dataset, and the designed derivatives. The resulting 2D and 3D binding interactions are presented in Figure 5.

Erlotinib, a well-established EGFR tyrosine kinase inhibitor used clinically against non-small cell lung cancer, specifically targets the EGFR signaling pathway. During docking, it formed a conventional hydrogen bond with residue Met769, supported by hydrophobic contacts with Leu694, Leu764, Lys721, Ala719, and Leu820. Two additional carbon-hydrogen bonds were established with Thr830 and Gln767, enhancing the stability of the complex.

Similarly, molecule 17 -the most active compound in the training set- displayed extensive binding interactions. It established five hydrogen bonds with Asp831, Leu764, Ala719, Thr766, and Glu738, along with hydrophobic interactions (π-alkyl and π-anion contacts) involving residues such as Phe699, Val702, Asp831, Ala719, and Lys721. An electrostatic interaction with Met742 further stabilized the binding conformation, underscoring its high affinity for the receptor.

The designed ligands (Pred65-Pred98) demonstrated binding energies between −7.9 and −10.8 kcal/mol (Table 6), outperforming both Erlotinib (−7.3 kcal/mol) and molecule 17 (−8.6 kcal/mol). Among them, Pred65 exhibited binding characteristics closely resembling those of Erlotinib and compound **17**, interacting with the same key residues within the active site. These amino acids appear to play a central role in activity enhancement, consistent with earlier findings [21,22]. Importantly, Pred65 displayed additional hydrophobic interactions compared to both Erlotinib and compound **17**, which contributed to its improved binding affinity and complex stability.

### 2.7. Molecular Dynamics (MD)

**Figure 6:**
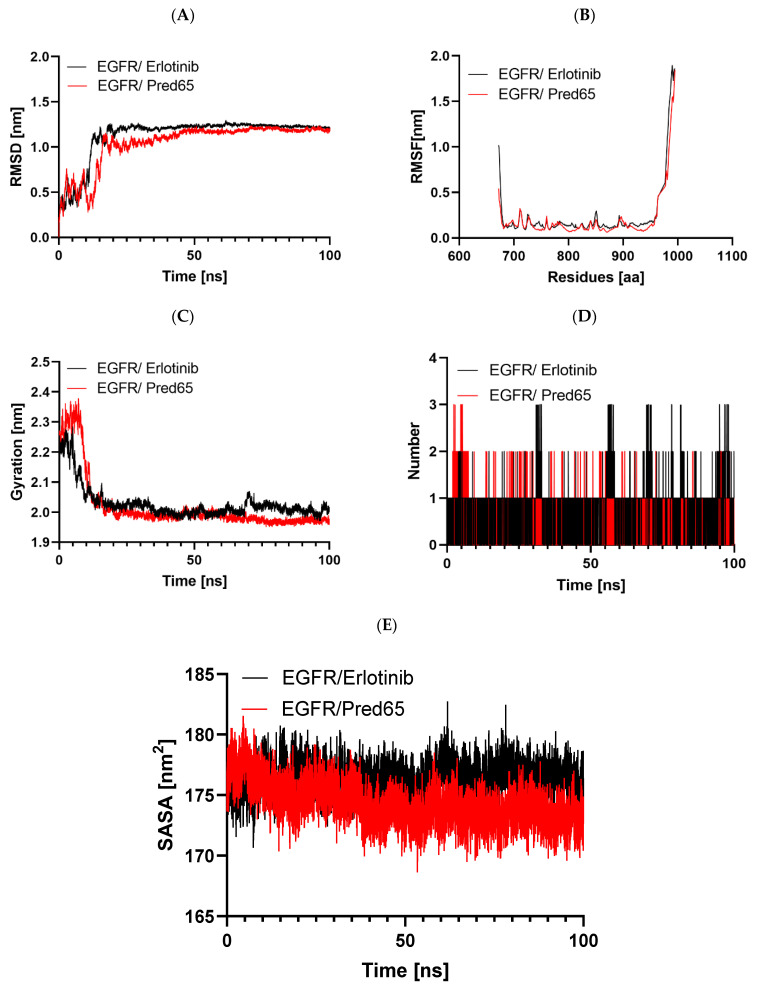
Comparative molecular dynamics evaluation of EGFR complexes with Erlotinib and Pred65: (**A**) backbone RMSD throughout the 100 ns simulation, (**B**) residue-level RMSF profiles, (**C**) changes in the radius of gyration (Rg), (**D**) hydrogen bond occupancy over time, and (**E**) solvent-accessible surface area (SASA) values.

Molecular dynamics (MD) simulations were conducted to assess the structural effects and stability of EGFR when complexed with the reference inhibitor Erlotinib and the designed compound Pred65, which demonstrated the strongest binding affinity (Table 6). The simulations were carried out using GROMACS 2019.3 (Figure 6). Trajectory analyses focused on key stability descriptors, including root mean square deviation (RMSD), root mean square fluctuation (RMSF), the radius of gyration (Rg), and Solvent Accessible Surface Area (SASA), providing detailed insights into the dynamic behavior of the protein–ligand complexes.

Pred65 was selected based on its superior docking score, favorable interaction profile, and acceptable ADMET properties. This targeted approach allows meaningful mechanistic insights while maintaining computational feasibility.

RMSD analysis

Protein conformational stability was assessed by monitoring RMSD throughout the simulations [23]. RMSD served as a principal metric for evaluating system convergence. For both the EGFR-Erlotinib and EGFR-Pred65 complexes, the trajectories over 100 ns showed no major deviations, indicating overall conformational stability (Figure 6A). In both systems, RMSD values increased from ~0.1 to 1.2 nm during the initial 0–15 ns, reflecting structural adjustments of the ligands within the binding site. Subsequently, the values stabilized, reaching a plateau near 1.0 nm. The relatively low RMSD values, particularly in the EGFR-Pred65 complex, confirm the robustness of the binding conformation and provide further support for the reliability of our docking and simulation results [9].

RMSF analysis

The RMSF analysis was used to evaluate the effect of ligand binding on the flexibility of EGFR residues. In this approach, higher RMSF values reflect greater residue mobility, while lower values correspond to increased rigidity. The RMSF profiles of the EGFR-Erlotinib and EGFR-Pred65 complexes are shown in Figure 6B. Overall, both complexes exhibited stable fluctuation patterns, with minimal variations in regions directly involved in ligand binding. The average RMSF values were 0.27 nm for EGFR-Erlotinib and 0.22 nm for EGFR-Pred65. These results indicate that Pred65 binding reduces the protein’s flexibility, thereby enhancing EGFR’s conformational stability.

Radius of gyration analysis

The Rg was analyzed to evaluate changes in the overall compactness of EGFR upon ligand binding. Typically, higher Rg values reflect greater structural flexibility and reduced stability, whereas lower values correspond to a more compact and stable protein conformation [24]. The mean Rg values were 2.3 nm for the EGFR-Erlotinib complex and 1.98 nm for the EGFR-Pred65 complex, suggesting that neither ligand induced major structural alterations in the protein. As illustrated in Figure 6C, the Rg of the EGFR-Pred65 complex reached equilibrium more rapidly during the 100-ns simulation, indicating superior conformational stability compared to the EGFR-Erlotinib complex.

Hydrogen bond analysis

Hydrogen bonding is a key determinant of ligand–protein interactions, strongly influencing binding affinity, specificity, metabolism, and absorption in drug discovery [25]. To evaluate the stability of the docked complexes, hydrogen bond formation was monitored for both EGFR-Erlotinib and EGFR-Pred65 systems in an explicit solvent environment during the MD simulations (Figure 6D). On average, Erlotinib maintained 0.96 hydrogen bonds within 0.35 nm of the binding pocket, whereas Pred65 exhibited a higher average of 1.3 hydrogen bonds under the same conditions. These results highlight stronger, more persistent hydrogen-bonding interactions between Pred65 and EGFR, supporting its superior binding stability compared to Erlotinib.

Solvent Accessible Surface Area (SASA) Analysis

The SASA reflects the portion of the protein surface that remains exposed to the surrounding solvent and therefore provides insight into protein compactness and solvent interaction [26]. In this study, SASA was tracked over a 100 ns molecular dynamics trajectory to assess how ligand binding influences the overall structural exposure of EGFR to water molecules.

As illustrated in the figure (Figure 6E), the EGFR–Erlotinib complex (black trace) maintains slightly elevated SASA values, averaging approximately 176–179 nm^2^, which indicates a relatively more solvent-exposed protein conformation. Conversely, the EGFR-Pred65 complex (red trace) consistently shows reduced SASA values of 171–175 nm2 throughout the simulation period. This persistent decrease suggests that Pred65 promotes a more compact arrangement of the protein, likely by enhancing intra-protein packing and stabilizing interactions within the active site. Notably, both complexes exhibit smooth, stable SASA profiles with no sudden fluctuations, indicating that ligand binding does not induce large-scale conformational disruption or unfolding. The lower solvent exposure observed for the Pred65-bound system further supports its stronger stabilizing effect on EGFR compared with the reference inhibitor.

MM-PBSA Binding Free Energy Analysis

The binding free energies of the EGFR complexes were estimated using the MM-PBSA approach, as implemented in the MmPbStat.py script [27], to evaluate the interaction strength of the reference inhibitor Erlotinib and the predicted compound Pred65. As summarized in Table 7, the EGFR/Pred65 complex exhibits a markedly more favorable total binding energy (−224.06 kJ/mol) compared with the EGFR/Erlotinib complex (−74.95 kJ/mol), indicating a significantly stronger predicted affinity of Pred65 toward EGFR.

Decomposition of the binding free energy components reveals distinct interaction profiles for the two ligands. In the EGFR/Erlotinib complex, binding stabilization is predominantly driven by van der Waals interactions, accompanied by a moderate electrostatic contribution. The SASA (non-polar solvation) term also contributes favorably (−20.89 ± 1.14 kJ/mol), supporting the role of hydrophobic interactions at the protein–ligand interface. In contrast, the EGFR/Pred65 complex exhibits a strongly favorable electrostatic energy (−509.84 kJ/mol), suggesting extensive charge-based interactions, such as salt bridges and stable hydrogen-bond networks, within the EGFR binding pocket. The van der Waals contribution for Pred65 (−53.68 ± 10.75 kJ/mol) is lower than that observed for Erlotinib, indicating that Pred65 binding relies more on electrostatic complementarity than on hydrophobic packing. The negative SASA energy (−11.12 ± 0.89 kJ/mol) further supports the presence of favorable non-polar interactions upon complex formation.

Overall, the MM-PBSA results suggest that Pred65 binds EGFR more strongly than the reference drug Erlotinib, with binding primarily governed by electrostatic interactions despite a higher desolvation cost. These findings are consistent with the enhanced structural stability observed during molecular dynamics simulations and support the potential of Pred65 as a promising EGFR inhibitor.

## 3. Materials and Methods

### 3.1. Data Set

Thirty-one quinazoline derivatives reported in the literature [28] were selected for QSAR analysis based on the availability of reliable experimental EGFR inhibitory activity measured under consistent assay conditions (Table 8). The dataset was divided into a training set of 26 compounds and an external test set of 5 compounds (**10**, **13**, **15**, **23**, and **34**) to evaluate model predictability.

The moderate dataset size reflects the limited number of structurally comparable quinazoline derivatives with homogeneous biological data suitable for robust 3D-QSAR modeling. Maintaining structural coherence is essential to minimize experimental noise and ensure meaningful correlations between molecular fields and biological activity, as commonly reported in CoMFA and CoMSIA studies on kinase inhibitors.

The training and test sets were generated using a rational structure–activity-based selection strategy rather than random splitting. The test set was chosen to cover the full range of biological activity and preserve structural diversity, ensuring an unbiased assessment of external predictive performance. Model robustness was further supported by cross-validation, external prediction, and established validation criteria.

### 3.2. Minimization and Alignment

Xiao and colleagues [29] have identified a considerable challenge in the alignment process used in CoMFA and CoMSIA studies. The SYBYL-X 2.0 software suite was employed to simulate the chemicals being analyzed. A module representing quinazoline variants was streamlined through the Tripos force field methodology [30]. Within the SYBYL framework, the assignment of Gasteiger–Huckel charges followed a strict convergence criterion of 0.01 kcal/mol. Figure 1 depicts the alignment of 31 quinazoline derivative molecules around a common core, with molecule 17, the most effective in the dataset, serving as the reference molecule.

### 3.3. Construction of the 3D-QSAR Model

Three-dimensional quantitative structure–activity relationship (3D-QSAR) methods have significantly influenced modern medicinal chemistry [31]. One of the pioneering techniques, Comparative Molecular Field Analysis (CoMFA), correlates molecular structures with biological activity by evaluating steric and electrostatic properties of ligands. The procedure involves sampling field values across a 3D lattice and aligning molecules to minimize root mean square deviations, ensuring consistent field comparisons. Statistical treatment of the data is carried out using partial least squares (PLS) regression combined with cross-validation, providing models with enhanced predictive reliability that can be visualized through contour maps of coefficients. An extension of this approach, Comparative Molecular Similarity Indices Analysis (CoMSIA), incorporates additional descriptors such as hydrogen bond donor, hydrogen bond acceptor, and hydrophobic interactions [32], yielding a more comprehensive model. To validate predictive performance, experimental pIC_50_ values must be compared with those predicted by the models, using a validation set distinct from the training compounds [33]. In this study, model robustness was confirmed, as reflected in the agreement between observed and predicted activities for the 26 compounds of the training set (Table 2). The best-performing CoMSIA model integrated four descriptors: steric, electrostatic, hydrophobic, and hydrogen bond donor fields.

### 3.4. Partial Least Squares (PLS) Analysis

The construction of the 3D-QSAR models was carried out using partial least squares (PLS) regression to investigate correlations between two sets of variables [34]: the CoMFA/CoMSIA descriptors and the experimental pIC_50_ values representing anticancer activity. The optimal number of latent components (N) [35] and the cross-validated correlation coefficient (Q^2^) was determined through the leave-one-out (LOO) validation procedure. In addition, non-cross-validated analyses were performed to calculate the coefficient of determination (R^2^), the F-test statistic, and the standard error of estimate (SEE) [36], ensuring accurate evaluation of model performance.

### 3.5. In Silico Pharmacokinetics ADMET Study

The effectiveness and viability of drug molecules hinge on their pharmacodynamic and pharmacokinetic characteristics, which include vital aspects such as high therapeutic effectiveness, binding affinity, permissible toxicity levels, and selectivity for the target protein [37]. This research utilized online platforms like pkCSM [10] and SwissADME [38] for assessing drug-like properties and the ADMET (absorption, distribution, metabolism, excretion, and toxicity) profiles. The approach involved employing in silico predictions to evaluate intestinal absorption, penetration of the blood–brain barrier into the central nervous system, biotransformation, clearance, and the genotoxic potential (AMES test) of prospective drug candidates [39].

### 3.6. Molecular Docking Studies

In this work, molecular docking experiments were carried out for eighteen ligands using AutoDock Vina https://vina.scripps.edu/downloads/ (accessed 30 December 2022) [40] in combination with AutoDock Tools [41] under a Windows 10 environment. The preparation of input files was performed using AutoDock Tools, in which ligand structures were converted from MOL format to PDBQT format. The epidermal growth factor receptor (EGFR) crystal structure (PDB ID: 1M17) [42] was obtained from the RCSB Protein Data Bank, selected for its high resolution and the relevance of its co-crystallized ligand to the compounds under investigation. Prior to docking, extraneous water molecules and ions were removed with Discovery Studio 2021 [43], and polar hydrogens were added to improve the accuracy of protein-ligand interactions. The docking grid was defined as a cubic box of 40 × 40 × 40 points in the XYZ directions, centered at coordinates 22.014, 0.253, and 52.794, with all parameters recorded in a configuration file (“conf.txt”). AutoDock’s scoring function generated nine binding poses for each ligand, ranked according to their binding free energy (ΔG) [20]. The conformations with the most favorable (lowest) ΔG values were retained as the most stable. Further analyses of hydrogen-bonding patterns and hydrophobic contacts were conducted in Discovery Studio to provide detailed insights into molecular interactions within the EGFR active site.

To validate the docking protocol, the co-crystallized ligand from the EGFR crystal structure (PDB ID: 1M17) was re-docked into the active site using the same docking parameters applied to the designed compounds. The re-docked pose exhibited an excellent overlap with the experimental binding conformation, yielding a root mean square deviation (RMSD) of 1.2 Å.

An RMSD value below 2.0 Å is generally considered indicative of a reliable docking protocol [44]. This result confirms the accuracy and robustness of the docking methodology employed in this study.

### 3.7. Molecular Dynamics (MD) Simulations

Molecular dynamics (MD) simulations were performed with GROMACS 2019.3 to investigate the structural dynamics of the top-ranked docking complexes, using the CHARMM27 force field [45]. Protein topologies were generated through the pdb2gmx module in GROMACS, based on the Chemistry at Harvard Macromolecular Mechanics (CHARMm ff) parameters [46], whereas ligand topologies were derived from the SwissParam server [47]. Each protein–ligand complex was inserted into a cubic simulation box of 9.6 nm per side and solvated with the TIP3P water model [48]. To ensure system neutrality, chloride and sodium ions were introduced as required. Energy minimization was conducted using the steepest descent method until the maximum force dropped below 1000 kJ/mol/nm. Equilibration was performed under constant pressure (1 bar) and temperature (300 K) conditions, using the Nose-Hoover thermostat and Parrinello-Rahman barostat [49]. Subsequently, 100-ns production simulations were executed. Post-simulation analyses were performed with standard GROMACS utilities, including RMSD (gmx rms), residue-level fluctuations (RMSF via gmx rmsf), radius of gyration (Rg), Solvent Accessible Surface Area (SASA), and hydrogen bond occupancy (gmx hbond) [50].

## 4. Conclusions

This study presents a comprehensive in silico exploration of quinazoline derivatives as potential EGFR inhibitors, employing a multi-modal approach that integrates 3D-QSAR modeling, ADMET profiling, molecular docking, and molecular dynamics simulations. Our findings demonstrate that, among the compounds studied, Pred65 emerged as a promising computational lead, exhibiting favorable predicted binding affinity, interaction patterns, and dynamic stability within the EGFR binding site compared to the reference inhibitor Erlotinib. These findings are based on in silico analyses and indicate the potential of Pred65 as a candidate for further experimental validation. Through rigorous molecular docking and MD analyses, Pred65 has shown strong interactions with critical residues in the EGFR binding pocket, underscoring its potential as a potent and selective EGFR inhibitor.

By leveraging computational techniques, this study offers valuable insights into the structural and pharmacokinetic properties essential for effective EGFR inhibition, highlighting the strengths of an in silico drug design approach. These findings not only pave the way for further experimental validation of Pred65 but also provide a framework for developing novel quinazoline-based EGFR inhibitors. Ultimately, this research contributes to advancing targeted therapies for lung cancer, supporting ongoing efforts to enhance therapeutic efficacy and address drug resistance in cancer treatment.

## Figures and Tables

**Table 1:** Statistical parameters of the developed CoMFA and CoMSIA models.

Generated Model	Q^2^	N	SEE	R^2^	F	R^2^_pred_	Fractions				
							S	E	H	D	A
CoMFA/SE	0.645	5	0.157	0.981	202.418	0.929	0.426	0.574			
CoMSIA/EHDA	0.710	7	0.175	0.979	117.142	0.956		0.375	0.240	0.229	0.155
**CoMSIA/SEHD**	**0.729**	**6**	**0.173**	**0.978**	**139.580**	**0.909**	**0.129**	**0.399**	**0.240**	**0.231**	
CoMSIA/EHD	0.652	7	0.179	0.977	111.520	0.981		0.438	0.280	0.282	
CoMSIA/SEH	0.702	7	0.178	0.978	112.816	0.982	0.185	0.482	0.333		

**Table 2:** Experimental pIC_50_ values and predicted pIC_50_ values obtained from the CoMSIA model, along with descriptor contributions (* test set).

Compounds	pIC_50_	pIC_50pred_	S	E	H	D
**1**	6.463	6.319	7.904	1.015	6.4348	1.5788
**2**	7.252	7.478	7.9196	0.9985	6.6941	1.5773
**3**	7.638	7.429	7.9325	0.9728	7.6152	1.5804
**4**	7.569	7.669	7.9393	0.9819	8.4325	1.5767
**6**	6.239	6.241	8.3975	1.2608	8.063	1.5964
**7**	7.260	7.281	8.4063	1.1907	5.8762	1.5788
**8**	7.523	7.57	8.4233	1.1889	8.0924	1.6007
**9**	6.114	6.318	8.1913	1.1001	5.7655	2.8167
**12**	5.301	5.288	8.2443	1.3587	6.1601	1.5839
**14**	6.921	7.004	8.3846	1.2338	5.8952	1.6015
**16**	7.000	7.133	8.193	1.1114	5.8389	2.7396
**17**	8.699	8.389	8.2065	1.0921	6.1384	2.7447
**21**	8.482	8.472	8.6549	1.3328	7.5729	2.7171
**22**	4.921	4.815	8.2492	1.324	6.1734	1.5745
**24**	6.092	6.017	8.2704	1.2904	7.3981	1.5758
**25**	6.000	6.211	8.2794	1.3026	8.2274	1.5681
**27**	7.538	7.420	8.9667	1.3337	5.5474	1.5486
**28**	8.42	8.564	8.9817	1.3155	5.8283	1.5433
**33**	8.398	8.142	8.6182	1.1087	8.0082	2.3807
**35**	7.921	7.925	8.9899	1.3085	8.1518	2.2802
**36**	8.328	8.272	8.0704	1.1116	8.1275	1.9375
**37**	7.398	7.44	8.8965	1.4343	8.1568	2.231
**38**	8.155	8.403	8.6457	1.1085	8.0595	2.2265
**39**	7.921	8.023	9.0264	1.1025	8.2194	2.2177
**40**	7.959	7.797	9.0698	1.1741	8.3894	1.5728
**43**	6.799	6.692	9.4074	1.2411	7.8388	2.838
10 *	6.241	7.472	8.666	1.3155	7.5086	2.8747
13 *	6.046	7.377	8.2733	1.1645	8.2324	1.582
15 *	8.000	8.315	8.4496	1.2035	8.0584	1.5705
23 *	5.215	6.869	8.2617	1.1676	6.4489	1.5757
34 *	7.076	8.303	9.0727	1.1625	8.407	1.5747

**Table 3:** Chemical structures and predicted pIC_50_ activities of the designed compounds based on the 3D-QSAR model.

N°	Structure	pIC_50_ (Pred)	
		CoMFA	CoMSIA/SEHD
**Pred65**	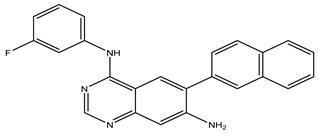	8.269	8.899
**Pred69**	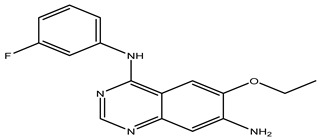	8.589	9.209
**Pred70**	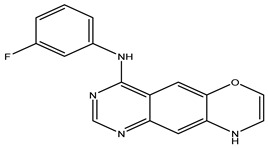	8.695	8.739
**Pred73**	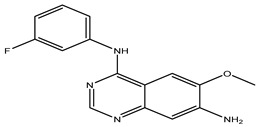	8.632	8.941
**Pred75**	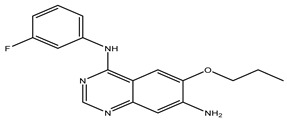	8.27	9.395
**Pred76**	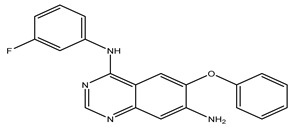	8.696	9.086
**Pred77**	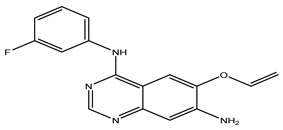	8.41	8.788
**Pred78**	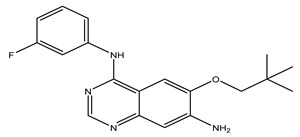	8.316	9.619
**Pred82**	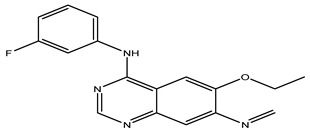	8.282	8.983
**Pred86**	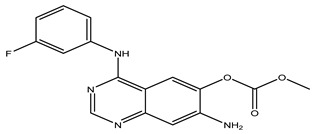	8.133	8.814
**Pred87**	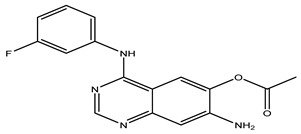	8.515	9.064
**Pred88**	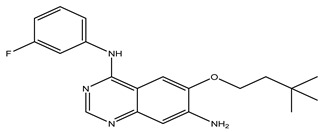	8.591	9.667
**Pred89**	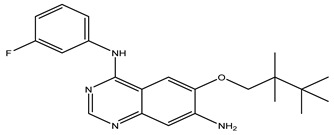	8.457	9.929
**Pred90**	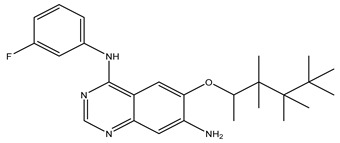	8.265	9.499
**Pred93**	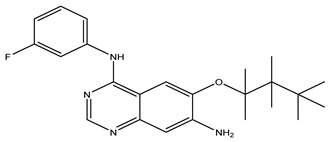	8.354	9.767
**Pred94**	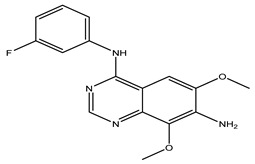	8.072	8.961
**Pred96**	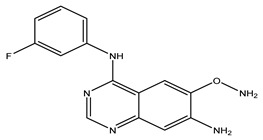	8.733	8.861
**Pred98**	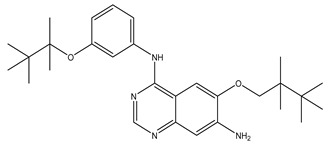	8.359	9.079

**Table 4:** Lipinski properties of the new designed quinazoline derivatives.

Inhibitor	Property								
	MW	LogP	NROT	NHA	NHD	TPSA	Lipinski’s Violations	Veber Violations	Egan Violations
Rule	<500	<=5	<10	<10	<5	<140	<=1	<=1	<=1
**Pred65**	380.426	5.59005	3	5	2	62.77	1	0	1
**Pred69**	298.321	2.71368	4	6	2	72.00	0	0	0
**Pred70**	294.289	2.84631	2	6	2	58.01	0	0	0
**Pred73**	284.107	2.28579	3	6	2	72.00	0	0	0
**Pred75**	312.138	3.05326	5	6	2	72.00	0	0	0
**Pred76**	346.122	3.87162	4	6	2	72.00	0	0	0
**Pred77**	296.107	2.74076	4	6	2	72.00	0	0	0
**Pred78**	340.169	3.7651	5	6	2	72.00	0	0	0
**Pred82**	310.122	3.15527	5	6	1	58.34	0	0	0
**Pred86**	328.097	2.36854	5	6	2	98.30	0	0	0
**Pred87**	312.102	2.17513	4	6	2	89.07	0	0	0
**Pred88**	354.185	4.74483	6	6	2	72.00	0	0	0
**Pred89**	382.216	5.45667	6	6	2	72.00	0	0	1
**Pred90**	438.279	7.61589	7	6	2	72.00	1	0	1
**Pred93**	410.248	6.3386	6	6	2	72.00	1	0	1
**Pred94**	314.320	2.18826	4	7	2	81.23	0	0	0
**Pred96**	285.102	0.959679	3	7	3	98.02	0	0	0
**Pred98**	478.330	7.55914	9	6	2	81.231	1	0	1

**Table 5:** ADMET properties of the new designed quinazoline derivatives.

Ligands Pred	Properties												
	Absorption	Distribution			Metabolism							Excretion	Toxicity
	Human Intestinal Absorption	VDss (Human)	BBB	CNS	Cytochrome P450 (CYP450)							Total Clearance	AMES Toxicity
					Substrate		Inhibitor						
					2D6	3A4	1A2	2C19	2C9	2D6	3A4		
	(%Absorbed)	log L/kg	log BB	log PS	Categorical (Yes/No)							log ml/min/k	Categorical (Yes/No)
**65**	92.799	−1.485	−0.001	−1.503	No	Yes	Yes	Yes	Yes	No	No	0.122	No
**69**	90.228	0.199	0.031	−2.273	No	Yes	Yes	Yes	Yes	No	Yes	0.04	No
**70**	89.523	0.252	−0.101	−2.036	No	Yes	Yes	Yes	Yes	Yes	Yes	−0.047	No
**73**	90.647	0.091	0.045	−2.265	No	Yes	Yes	Yes	Yes	No	Yes	0.006	No
**75**	90.079	0.159	0.031	−2.252	No	Yes	Yes	Yes	No	No	Yes	0.055	No
**76**	90.248	−0.989	0.127	−1.931	No	Yes	Yes	Yes	Yes	No	Yes	−0.041	No
**77**	89.911	0.2	0.032	−2.248	No	Yes	Yes	Yes	Yes	No	Yes	0.022	No
**78**	89.355	−0.12	0.136	−1.912	No	Yes	Yes	Yes	Yes	No	Yes	−0.072	No
**82**	91.68	0.003	−0.237	−2.152	No	Yes	Yes	Yes	Yes	Yes	No	0.025	No
**86**	100	−0.129	−0.289	−3.115	No	Yes	Yes	Yes	Yes	No	Yes	−0.255	No
**87**	90.791	−0.011	−0.121	−2.346	No	Yes	Yes	Yes	Yes	No	No	0.017	No
**88**	88.806	−0.092	0.153	−1.899	No	Yes	Yes	Yes	Yes	No	Yes	−0.052	No
**89**	88.357	0.005	0.183	−1.746	No	Yes	Yes	Yes	Yes	No	Yes	−0.044	No
**90**	87.41	0.182	0.202	−1.495	No	Yes	No	Yes	Yes	No	Yes	0.01	No
**93**	89.353	0.273	0.205	−1.57	No	Yes	Yes	Yes	Yes	No	Yes	0.017	No
**94**	90.455	−0.271	−0.634	−2.396	No	Yes	Yes	Yes	Yes	No	Yes	−0.075	No
**96**	91.732	0.368	−0.971	−2.521	No	No	Yes	No	No	No	No	−0.06	No
**98**	87.034	−0.161	0.06	−1.45	No	Yes	No	Yes	Yes	No	Yes	−0.272	No

**Table 6:** Binding Affinity Results for the Most Stable Conformation.

Compound	Binding Affinity (kcal/mol)
Erlotinib	−7.3
Molecule 17	−8.6
Pred65	−10.8
Pred69	−8
Pred70	−9.2
Pred73	−8.2
Pred75	−8.1
Pred76	−9.1
Pred77	−8
Pred78	−8.5
Pred82	−8.3
Pred86	−8.3
Pred87	−8.4
Pred88	−8.1
Pred89	−8.5
Pred90	−9.1
Pred93	−9
Pred94	−7.9
Pred96	−8.1
Pred98	−9.1

**Table 7:** MM-PBSA calculations of binding free energy for the selected complexes.

Complexes	Binding Energy (kJ/mol)	SASA Energy (kJ/mol)	Polar Solvation Energy (kJ/mol)	Electrostatic Energy (kJ/mol)	Van Der Waal Energy (kJ/mol)
EGFR/Erlotinib	−74.945 +/− 13.761	−20.885 +/− 1.143	173.553 +/− 21.470	−40.552 +/− 13.997	−187.061 +/− 11.334
EGFR/Pred65	−224.059 +/− 23.272	−11.119 +/− 0.892	350.581 +/− 41.796	−509.843 +/− 43.616	−53.678 +/− 10.752

**Table 8:** pIC_50_ values of the reported quinazoline derivatives against tyrosine kinase (EGFR) (* test set).

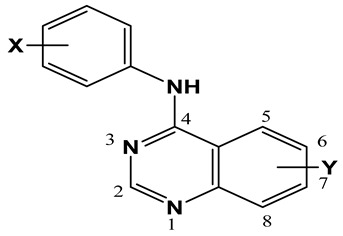			
No.	Substituent		pIC_50_
	X	Y	
1	H	H	6.463
2	3-F	H	7.252
3	3-Cl	H	7.638
4	3-Br	H	7.569
6	3-CF_3_	H	6.239
7	H	6-OMe	7.260
8	3-Br	6-OMe	7.523
9	H	6-NH_2_	6.114
10 *	3-CF_3_	6-NH_2_	6.241
12	H	6-NO_2_	5.301
13 *	3-Br	6-NO_2_	6.046
14	H	7-OMe	6.921
15 *	3-Br	7-OMe	8.000
16	H	7-NH_2_	7.000
17	3-F	7-NH_2_	8.699
21	3-CF_3_	7-NH_2_	8.482
22	H	7-NO_2_	4.921
23 *	3-F	7-NO_2_	5.215
24	3-Cl	7-NO_2_	6.091
25	3-Br	7-NO_2_	6.000
27	H	6,7-Di-OMe	7.538
28	3-F	6,7-Di-OMe	8.420
33	3-Br	6-NHMe	8.398
34 *	3-Br	6-NMe_2_	7.076
35	3-Br	6-NHCOOMe	7.921
36	3-Br	7-OH	8.328
37	3-Br	7-NHCOMe	7.398
38	3-Br	7-NHMe	8.155
39	3-Br	7-NHC_2_H_5_	7.921
40	3-Br	7-NMe_2_	7.959
43	3-Br	6-NH_2_,7-NMe_2_	6.799

## Data Availability

All essential data supporting the findings of this study are included in the article. Additional large raw computational files (docking poses, scoring files, and full molecular dynamics trajectories) are available from the corresponding author upon reasonable request.
